# Supplementary material for: Bayesian refinement of protein structures and ensembles against SAXS data using molecular dynamics
Source: PLoS Comput Biol. 2017 Oct 18;13(10):e1005800. doi: 10.1371/journal.pcbi.1005800 (PMC5662244; doi:10.1371/journal.pcbi.1005800)
Supplement: S1 Archive — (BZ2) [file pcbi.1005800.s011.tar.bz2 › gromacs_4.62_bayesian_saxs_refi/share/html/online/g_chi.html]

g\_chi


| g\_chi Main Table of Contents |

**VERSION 4.6  
Sat 19 Jan 2013**

---

### Description

g\_chi computes φ, ψ, ω, and χ dihedrals for all your
amino acid backbone and sidechains.
It can compute dihedral angle as a function of time, and as
histogram distributions.
The distributions (histo-(dihedral)(RESIDUE).xvg) are cumulative over all residues of each type.

If option -corr is given, the program will
calculate dihedral autocorrelation functions. The function used
is C(t) = . The use of cosines
rather than angles themselves, resolves the problem of periodicity.
(Van der Spoel & Berendsen (1997), Biophys. J. 72, 2032-2041).
Separate files for each dihedral of each residue
(corr(dihedral)(RESIDUE)(nresnr).xvg) are output, as well as a
file containing the information for all residues (argument of -corr).

With option -all, the angles themselves as a function of time for
each residue are printed to separate files (dihedral)(RESIDUE)(nresnr).xvg.
These can be in radians or degrees.

A log file (argument -g) is also written. This contains   
(a) information about the number of residues of each type.  
(b) The NMR ^3J coupling constants from the Karplus equation.  
(c) a table for each residue of the number of transitions between
rotamers per nanosecond, and the order parameter S^2 of each dihedral.  
(d) a table for each residue of the rotamer occupancy.

All rotamers are taken as 3-fold, except for ω and χ dihedrals
to planar groups (i.e. χ\_2 of aromatics, Asp and Asn; χ\_3 of Glu
and Gln; and χ\_4 of Arg), which are 2-fold. "rotamer 0" means
that the dihedral was not in the core region of each rotamer.
The width of the core region can be set with -core\_rotamer

The S^2 order parameters are also output to an .xvg file
(argument -o ) and optionally as a .pdb file with
the S^2 values as B-factor (argument -p).
The total number of rotamer transitions per timestep
(argument -ot), the number of transitions per rotamer
(argument -rt), and the ^3J couplings (argument -jc),
can also be written to .xvg files. Note that the analysis
of rotamer transitions assumes that the supplied trajectory frames
are equally spaced in time.

If -chi\_prod is set (and -maxchi > 0), cumulative rotamers, e.g.
1+9(χ\_1-1)+3(χ\_2-1)+(χ\_3-1) (if the residue has three 3-fold
dihedrals and -maxchi >= 3)
are calculated. As before, if any dihedral is not in the core region,
the rotamer is taken to be 0. The occupancies of these cumulative
rotamers (starting with rotamer 0) are written to the file
that is the argument of -cp, and if the -all flag
is given, the rotamers as functions of time
are written to chiproduct(RESIDUE)(nresnr).xvg
and their occupancies to histo-chiproduct(RESIDUE)(nresnr).xvg.

The option -r generates a contour plot of the average ω angle
as a function of the φ and ψ angles, that is, in a Ramachandran plot
the average ω angle is plotted using color coding.

### Files

| option | filename | type | description |
| --- | --- | --- | --- |
| **-s** | conf.gro | Input | Structure file: gro g96 pdb tpr etc. |
| **-f** | traj.xtc | Input | Trajectory: xtc trr trj gro g96 pdb cpt |
| **-o** | order.xvg | Output | xvgr/xmgr file |
| **-p** | order.pdb | Output, Opt. | Protein data bank file |
| **-ss** | ssdump.dat | Input, Opt. | Generic data file |
| **-jc** | Jcoupling.xvg | Output | xvgr/xmgr file |
| **-corr** | dihcorr.xvg | Output, Opt. | xvgr/xmgr file |
| **-g** | chi.log | Output | Log file |
| **-ot** | dihtrans.xvg | Output, Opt. | xvgr/xmgr file |
| **-oh** | trhisto.xvg | Output, Opt. | xvgr/xmgr file |
| **-rt** | restrans.xvg | Output, Opt. | xvgr/xmgr file |
| **-cp** | chiprodhisto.xvg | Output, Opt. | xvgr/xmgr file |

### Other options

| option | type | default | description |
| --- | --- | --- | --- |
| **-[no]h** | bool | no | Print help info and quit || **-[no]version** | bool | no | Print version info and quit || **-nice** | int | 19 | Set the nicelevel || **-b** | time | 0 | First frame (ps) to read from trajectory || **-e** | time | 0 | Last frame (ps) to read from trajectory || **-dt** | time | 0 | Only use frame when t MOD dt = first time (ps) || **-[no]w** | bool | no | View output .xvg, .xpm, .eps and .pdb files || **-xvg** | enum | xmgrace | xvg plot formatting: xmgrace, xmgr or none || **-r0** | int | 1 | starting residue || **-[no]phi** | bool | no | Output for φ dihedral angles || **-[no]psi** | bool | no | Output for ψ dihedral angles || **-[no]omega** | bool | no | Output for ω dihedrals (peptide bonds) || **-[no]rama** | bool | no | Generate φ/ψ and χ\_1/χ\_2 Ramachandran plots || **-[no]viol** | bool | no | Write a file that gives 0 or 1 for violated Ramachandran angles || **-[no]periodic** | bool | yes | Print dihedral angles modulo 360 degrees || **-[no]all** | bool | no | Output separate files for every dihedral. || **-[no]rad** | bool | no | in angle vs time files, use radians rather than degrees. || **-[no]shift** | bool | no | Compute chemical shifts from φ/ψ angles || **-binwidth** | int | 1 | bin width for histograms (degrees) || **-core\_rotamer** | real | 0.5 | only the central -core\_rotamer\*(360/multiplicity) belongs to each rotamer (the rest is assigned to rotamer 0) || **-maxchi** | enum | 0 | calculate first ndih χ dihedrals: 0, 1, 2, 3, 4, 5 or 6 || **-[no]normhisto** | bool | yes | Normalize histograms || **-[no]ramomega** | bool | no | compute average omega as a function of φ/ψ and plot it in an .xpm plot || **-bfact** | real | -1 | B-factor value for .pdb file for atoms with no calculated dihedral order parameter || **-[no]chi\_prod** | bool | no | compute a single cumulative rotamer for each residue || **-[no]HChi** | bool | no | Include dihedrals to sidechain hydrogens || **-bmax** | real | 0 | Maximum B-factor on any of the atoms that make up a dihedral, for the dihedral angle to be considere in the statistics. Applies to database work where a number of X-Ray structures is analyzed. -bmax <= 0 means no limit. || **-acflen** | int | -1 | Length of the ACF, default is half the number of frames || **-[no]normalize** | bool | yes | Normalize ACF || **-P** | enum | 0 | Order of Legendre polynomial for ACF (0 indicates none): 0, 1, 2 or 3 || **-fitfn** | enum | none | Fit function: none, exp, aexp, exp\_exp, vac, exp5, exp7, exp9 or erffit || **-ncskip** | int | 0 | Skip this many points in the output file of correlation functions || **-beginfit** | real | 0 | Time where to begin the exponential fit of the correlation function || **-endfit** | real | -1 | Time where to end the exponential fit of the correlation function, -1 is until the end |

### Known problems

- Produces MANY output files (up to about 4 times the number of residues in the protein, twice that if autocorrelation functions are calculated). Typically several hundred files are output.- φ and ψ dihedrals are calculated in a non-standard way, using H-N-CA-C for φ instead of C(-)-N-CA-C, and N-CA-C-O for ψ instead of N-CA-C-N(+). This causes (usually small) discrepancies with the output of other tools like g\_rama.- -r0 option does not work properly- Rotamers with multiplicity 2 are printed in chi.log as if they had multiplicity 3, with the 3rd (g(+)) always having probability 0

---

http://www.gromacs.org  
gromacs@gromacs.org
